# Mismatch negativity in patients with (central) auditory processing disorders

**DOI:** 10.1016/S1808-8694(15)31380-X

**Published:** 2015-10-17

**Authors:** Simone Mariotto Roggia, Nádia Tenório Colares

**Affiliations:** 1 Doctor, adjunct professor of the Speech Therapy course, UNIVALI - SC; 2 Student of Speech Therapy, UNIVALI - SC; science initiation scholarship. Universidade do Vale do Itajaí

**Keywords:** hearing, auditory evoked potentials, hearing disorders

## Abstract

Mismatch Negativity (MMN) is a long-latency auditory evoked potential that provides an objective index of discrimination skills and auditory sensorial memory. It may, therefore, be used as an electrophysiological evaluation of central auditory processing.

**Aim:**

To study MMN in patients with Central Auditory Processing Disorder - CAPD.

**Study Method:**

A prospective clinical study.

**Material and Method:**

Eight individuals with CAPD, aged between nine and 14 years, were evaluated; there was also a control group. MMN was elicited for tone stimuli (tone bursts), differing in terms of frequency (MMNf - standard stimulus: 750 Hz and deviant stimulus: 1,000 Hz), as well as duration (MMNd - standard stimulus: 100 ms and deviant stimulus: 50 ms; at 1,000 Hz).

**Results:**

The presence of MMNf and of MMNd was statistically demonstrated in both groups. No significant statistical differences, however, were observed between MMNf and MMNd latencies and amplitude values in the two groups. Also, no significant statistical differences were observed between the MMNf and the MMNd among the groups.

**Conclusion:**

The CAPD individuals that were evaluated showed no changes in MMNf or MMNd.

## INTRODUCTION

Central auditory processing may be defined as the events that occur when the brain recognizes and interprets sounds around a person.^1^

It consists of a set of auditory abilities that are used to understand speech information, such as: the ability to detect and locate sounds spatially, sound discrimination and recognition, selective and sustained attention to sound, and the ability to retain these sounds.^1^, ^2^

Central auditory processing disorders - (C)APD - are difficulties in the processing of perception of auditory information by the central nervous system; it is demonstrated by a poor performance in one or more auditory processing abilities,^3^ including sound location and lateralization, auditory discrimination, recognition of auditory patterns, temporal aspects of audition, auditory performance in the presence of competing acoustic signals and auditory performance of degraded auditory signals.^4^

Generally, children with (C)APD may present the following: in general, these children are male, aged between six and eight years, with learning disabilities especially with reading, intelligence within normal limits, often with left or mixed hand dominance, normal fine and coarse motor abilities, occasional delayed speech acquisition, and no evidence of neurological damage or signs.^5^

The assessment of central auditory processing may be done by behavioral and electrophysiological auditory tests. Behavioral auditory tests are the most commonly used clinically, but their efficacy has been questioned in recent years, since non-auditory factors - such as attention and motivation - may interfere with these tests.^6^

The inclusion of electrophysiological measurements in assessing the central nervous system is at times essential; in certain cases, electrophysiological tests are superior to behavioral tests in identifying central auditory nervous system dysfunction.^7^ Furthermore, electrophysiological tests are less affected by irrelevant variables.^8^ Electrophysiological tests for assessing auditory processing include the brainstem auditory evoked potentials (BAEP), middle latency responses, N1 and P2 responses, the P300, and Mismatch Negativity (MMN).^9^

The MMN is an electrical brain response elicited by any type of discriminated change in any repetitive aspect of auditory stimulation.^10^ It is an auditory evoked potential (AEP) characterized as a negative wave that is independent of the subject’s attention, and that originates mainly in the auditory cortex.^11^ The MMN reflects automatic pre-attention auditory discrimination and the activation of echoic memory.^12^ It is the subtraction of auditory event-related potentials (AERP) obtained for a standard stimulus minus the AERP obtained for the deviant stimulus.^13^

It is usually obtained using an oddball paradigm in which a repetitive standard stimulus is occasionally replaced by a deviant stimulus.^13^ It may be elicited not only by pure tones and their elementary variants (frequency, duration, intensity, direction, etc.) but also by complex stimuli, including speech.^12^

An important feature of these AEPs are the fact that they are generated independently of the subject’s attention,^11^, ^14^, ^15^ making it a valuable objective measure of auditory discrimination.^10^, ^15^

Although there have been many published studies on MMN, up to the present there is a single case study^17^ in which MMN was investigated in a patient with (C)APD and no other associated conditions. In this study verbal stimuli were applied to elicit MMN, which were absent for the stimuli that were used.

Another study^18^ also investigated MMN in patients with suspected (C)APD; these subjects had been identified in screening tests for (C)AP although a diagnosis had not been confirmed by behavioral assessments. In this study, MMN was normal for speech stimuli deviating in terms of the articulation point.^18^

The investigation of MMN in (C)APD patients with associated comorbidities due to other diseases shows that MMN was altered for speech stimuli but normal for pure tone stimuli in some studies,^19^, ^20^, ^21^, ^22^ that MMN was altered for verbal and non-verbal stimuli in other studies,^23^ still others in which MMN was altered for pure tone stimuli with frequency deviations,^24^, ^25^ studies in which MMN was altered for pure tones with frequency but not duration deviations,^26^, ^27^ studies in which MMN was normal for pure tones with frequency deviations but altered for different compound tone patters,^28^ and in studies in which MMN was unchanged for frequency and duration stimuli.^29^, ^30^, ^31^, ^32^

Our study aimed to investigate MMN in (C)APD patients, considering the diversity of results in studies of MMN in these patients, as well as the importance of undertaking the electrophysiological evaluation of auditory processing studies.

## MATERIAL AND METHOD

The Research Ethics Committee of the university in which the study was done analyzed this study, which involved human beings, and approved it on 12 July 2006 (protocol number 278/06).

Our series consisted of eight subjects (experiment group) with a diagnosis of (C)APD based on behavioral evaluations of auditory processing done in 2006 at the Audiology Clinic of the university and eight normal hearing children (control group). Both groups were matched for sex, age and social/economic level. The age ranged from nine to 14 years, and each groups contained four male and four female subjects.

Parents or caretakers were invited and informed about the aims of this study; those that agreed to allow their children to participate signed a free informed consent form.

The following inclusion criteria were used: bilateral auditory thresholds within normal limits at 15 dBNA (Northern and Downs - for the evaluation of children);^33^ a speech recognition threshold (SRT) within the tone average, that is, equal to mean values of 500, 1000 and 2000 Hz or up to 10 dB worse;^34^ a speech recognition index (SRI) over 88%;^35^ and type A tympanometric curves.^36^

For the control group only subjects with no clinical intercurrences that might affect the central auditory nervous system were selected; these patients had a history of not more than three episodes of otitis during the language development phase, no delayed neuropsychomotor development, no clinical events in pregnancy or delivery, and no school complaints such as hyperactive behavior or lack of attention. The control group was selected based on files of patients seen at the Audiology Clinic.

The digit dichotic test (test version on a CD recorded by Pereira and Schochat^37^) was applied to exclude patients with altered auditory processing in the control group; this test had to be normal according to the Santos^38^ criteria for subjects to be included as controls.

MMN was recorded in acoustically isolated rooms in the Audiology Clinic of the university; a model EP 25 (Interacoustics) device was used for measuring auditory evoked potentials.

Four disposable electrodes were placed in Fz (according to the international 10–20 system), in the right and left mastoids, and the left frontal area of the patient. The Fz position was chosen as it has been considered as the best for measuring MMN.^39^ The left mastoid electrode was used as a reference and the frontal area for the ground electrode. The right mastoid electrode was used for obtaining the necessary impedance for the test. Impedance was kept below 5 KW.

MMN was evaluated twice; it was first elicited for tone burst frequency deviant stimuli (standard stimulus: 750Hz; deviating stimulus: 1000Hz), which was named MMNf, after which MMN was elicited for duration deviant stimuli (standard stimulus: 100ms; deviating stimulus: 50ms, both at 1000Hz), which was named MMNd.

Frequency and duration deviant stimuli were presented randomly, in an oddball paradigm, at 1.3 stimuli per second, with a 20% probability. There were 800 stimuli presented to obtain at least 150 deviant promediated stimuli. Polarity was the alternate type.

Auditory stimuli were presented at 60 dBNA (auditory level) using 3A insertion phones coupled to the left ear; the aim was to use tone burst stimuli for eliciting MMNs, considering a right hemispheric dominance for this type of stimulus.

During MMN recording, subjects remained seated comfortably in passive hearing, watching a video for children; they were asked to remain as quiet as possible and to pay attention only to the film, rather than the stimuli. The film audio was kept at usual levels, but below 40 dBNPS (A scale).^14^, ^12^

AEPs were acquired, amplified, digitalized, promediated only, and filtered by a 25 Hz low-pass filter and a 1.67Hz high-pass filter. The recording window was 60ms prior to stimulation and 480ms after stimulation.^12^

MMN was identified as the highest negative peak within a 150 to 350ms40 time period, visualized in the deviant wave. The device calculated automatically the MMN amplitude and latency after the examiner had placed the cursor appropriately.

MMNs were recorded and analyzed in all control and experiment group subjects, after which mean latency and amplitude MMNf and MMNd values were calculated and analyzed statistically.

The t test for a sample was used to confirm statistically the presence of MMN and to verify if mean MMN amplitudes in each group, for each type of stimuli (frequency and duration deviation), were significantly different from zero.

The t test for comparing independent samples was applied to verify the presence of statistically significant differences between mean latency and amplitude values in both groups for the MMNf and MMNd. The aim was to check MMN duration or frequency differences between both groups.

The t test for comparing two dependent samples was applied to verify statistically significant differences between mean MMN latency and amplitude values obtained for frequency (MMNf) and duration (MMNd) within each group. The aim of this analysis as to check whether the eliciting MMN stimulus -frequency or duration deviations - yielded different MMNs in each group.

## RESULTS

As the sample was small (n = 8), we first applied the Kolmogorov-Smirnov test to verify it the samples originated from normal populations (5% significance). The normal hypothesis was not discarded in the samples, which allowed us to apply Student’s t test.

The mean MMNf and MMNd amplitude values and standard deviations for the control and experiment groups may be seen in Table 1, which also shows the results obtained by applying Student’s t test to verify whether the MMNf and MMNd amplitudes were null.

**Table 1:** Results of the statistical analysis (Student’s t test) on the variable amplitude.

STATISTICS (Amplitude)	CONTROL GROUP		EXPERIMENT GROUP	
	MMNf	MMNd	MMNf	MMNd
Mean	-1,96 mn	-1,61 mn	-1,70 mn	-2,07 mn
Standard deviation	0,481 mn	0,831 mn	1,146 mn	1,438 mn
t statistics	-11,5114	-5,48853	-4,20343	-4,07089
p-value	0,000008*	0,000918*	0,004018*	0,004744*

* Statistically significant difference

Table 1 shows that the presence of MMNf and MMNd was confirmed statistically at a 1% (a = 0.01) significance level in both groups.

Mean values and standard deviations for MMNf and MMNd latencies in the control and experiment groups may be seen in Table 2.

**Table 2:** Means and standard deviations for MMNf and MMN latencies in the control and experiment groups.

STATISTICS (Latency)	CONTROL GROUP		EXPERIMENT GROUP	
	MMNf	MMNd	MMNf	MMNd
Mean	220,00ms	219,25ms	225,25ms	245,25ms
Standard deviation	55,457ms	60,408ms	45,279ms	61,038ms

Statistical results obtained by comparing MMNf and MMNd latency and amplitude values in the control group and the experiment group may be seen in Table 3.

**Table 3:** Student’s t test (independent samples) for testing the differences between MMNf and MMNd latency and amplitude values in the control and experiment groups

CONTROL GROUP X EXPERIMENT GROUP	t statistics	p
MMNf latency	-0,207410	0,838676
MMNd latency	-0,856339	0,406229
MMNf amplitude	-0,582078	0,569770
MMNd amplitude	0,777163	0,449996

Table 3 shows that there was no statistically significant difference (5% significance level; a = 0.05) among latency and amplitude values in children with APD and the control group for both MMNf and MMNd.

Table 4 shows the results of the statistical analysis comparing MMN latency and amplitude values for frequency deviations (MMNf) and the elicited MMN for duration deviations (MMNd) in each group.

**Table 4:** Student’s t test (dependent samples) for testing MMNf and MMNd latency and amplitude value differences within the same group.

MMNf × MMNd	t statistics	p
Control group latency	0,024857	0,980863
Experiment group latency	-0,848080	0,424440
Control group amplitude	-1,21623	0,263312
Experiment group amplitude	0,678144	0,519461

Table 4 shows that there were no statistically significant differences among latency and amplitude values that depended on the MMN eliciting stimulus (frequency or duration) in either group.

## DISCUSSION

As MMN has a relatively low signal to noise ratio,^16^ demonstrating statistically its existence - verifying that its amplitude is significantly different from zero - has been associated with visual identification.^14^, ^41^, ^42^, ^43^, ^44^, ^45^, ^46^, ^47^, ^48^ Such statistical proof is generally done with a t test based on the mean MMN amplitude in subjects. Table 1 thus shows that MMNs elicited for duration and frequency deviations were statistically demonstrated in the experiment group - (C)APD - and the control group.

MMN is an electrical brain response elicited by any discriminated change in any repetitive aspect of auditory stimulation,^10^ arising independently of the subject’s attention.^11^ It thus may be concluded that both (C)APD subjects and the control group discriminated, at a pre-attention level - duration and frequency deviations of the stimuli.

MMN was demonstrated statistically in both study groups; however, there were no MMNf and MMNd latency and amplitude differences between the experiment - (C)APD - and control groups (Table 3). As in Liasis et al.’s1^8^ study, MMN could not be considered as a measure of the presence or absence of hearing disorders in (C)APD subjects.

A few hypotheses are raised here to explain these results.

The first concerns the number of subjects in our study; since our sample was small, the difference among MMNf and MMNd latency and amplitude values would have to be very large for a statistically significant difference to manifest. This does not seem plausible to us, however, as most of the studies using MMN have usually included about 10 subjects in each group.

A second hypothesis is that (C)APD subjects in this study had no frequency deviating pure tone discrimination problems, as was found in Meng et al.’s study,^28^ or duration deviating problems, as seen in Baldeweg et al.’s^26^ and Korpilahti and Lang’s studies.^27^ Or these subjects might have altered MMN only for verbal sounds, as shown in studies by Schulte-Körne et al.,^19^, ^20^ Uwer, Albrecht and Suchodoletz,^21^ and Sharma et al.^-^

We believe, therefore, that the lack of MMN changes in our study may be due to the fact that (C) APD subjects had auditory discrimination difficulties limited to acoustic parameters not assessed in our study.

The statistical analysis showed that MMN elicited for frequency deviating stimuli was similar to MMN elicited for duration deviating stimuli in the group of (C)APD subjects and controls (Table 4). This finding may suggest that the (C)APD subjects had difficulties in discriminating the frequency and duration deviations presented in our study.

It should be borne in mind, however, that our results should not be taken as indicating that (C) APD subjects have no type of duration or frequency discrimination changes, as we used only one type of frequency and one type of duration distinction for eliciting MMN. Thus, difficulties in discriminating the frequency or duration of more similar tone bursts than those used in this study cannot be discarded.

## CONCLUSION

(C)APD subjects in this study presented no MMN latency or amplitude changes elicited for frequency and duration stimuli.
